# Acute aortic catastrophe caused by cardiovascular oncological manipulation by tyrosine kinase inhibitors with immune checkpoint blockades: a case report and literature review

**DOI:** 10.1093/ehjcr/ytae169

**Published:** 2024-04-05

**Authors:** Sherif Sultan, Yogesh Acharya, Paul Donnellan, Niamh Hynes, Michael Kerin, Osama Soliman

**Affiliations:** Department of Vascular and Endovascular Surgery, Western Vascular Institute, University Hospital Galway, University of Galway, Newcastle Rd, Galway H91 YR71, Ireland; Department of Vascular and Endovascular Surgery, Galway Clinic, and Royal College of Surgeons in Ireland and University of Galway, Galway Affiliated Hospital, Doughiska Rd, Galway H91 HHT0, Ireland; CORRIB-CURAM-Vascular Group, University of Galway, Newcastle Rd, Galway H91 YR71, Ireland; Department of Cardiovascular Oncology, University Hospital Galway, University of Galway, Newcastle Rd, Galway H91 YR71, Ireland; Department of Vascular and Endovascular Surgery, Western Vascular Institute, University Hospital Galway, University of Galway, Newcastle Rd, Galway H91 YR71, Ireland; Department of Vascular and Endovascular Surgery, Galway Clinic, and Royal College of Surgeons in Ireland and University of Galway, Galway Affiliated Hospital, Doughiska Rd, Galway H91 HHT0, Ireland; Department of Oncology, University Hospital Galway, University of Galway, Newcastle Rd, Galway H91 YR71, Ireland; CORRIB-CURAM-Vascular Group, University of Galway, Newcastle Rd, Galway H91 YR71, Ireland; Department of Cardiovascular Oncology, University Hospital Galway, University of Galway, Newcastle Rd, Galway H91 YR71, Ireland; CORRIB-CURAM-Vascular Group, University of Galway, Newcastle Rd, Galway H91 YR71, Ireland; Department of Cardiovascular Oncology, University Hospital Galway, University of Galway, Newcastle Rd, Galway H91 YR71, Ireland

**Keywords:** Acute aortic syndrome, Aortic intra-mural haematoma, Tyrosine kinase inhibitors, Immune checkpoint blockers, Clear cell renal cell carcinoma, Case report

## Abstract

**Background:**

Tyrosine kinase inhibitors targeting the vascular endothelial growth factor (VEGF) inhibitor pathway with immune checkpoint blockade have shown promising outcomes in managing metastatic renal cancer. However, they increase the risk of a person developing high blood pressure and cardiovascular complications.

**Case summary:**

In this study, we report the case of a 73-year-old woman on axitinib and pembrolizumab for her Stage 4 renal cell carcinoma. She presented with intractable chest pain and high systolic blood pressure, not responding to opiates. Her computed tomography angiography results showed an acute intra-mural haematoma with a rupture in the descending thoracic aorta. She underwent emergency thoracic endovascular aortic repair. Post-operatively, she recovered fully without any neurological or cardiovascular issues.

**Discussion:**

The severity of cardiovascular haemodynamic complications arising from the consumption of VEGF inhibitors and from immunotherapy and the lack of anti-hypertensive strategies to adequately manage such events require an unequivocal and urgent assessment of their cardiovascular safety. This case highlights the crucial role of cardiovascular oncology in managing such acute aortic catastrophes.

**Learning points**

- A combination of tyrosine kinase inhibitors that target the vascular endothelial growth factor (VEGF) inhibitor pathway with immune checkpoint blockade has shown promising outcomes in metastatic renal cancer; however, they are associated with an increased risk of a person developing high blood pressure and cardiovascular complications, including acute aortic syndromes.
- It is important to be aware of the severity of cardiovascular haemodynamic complications arising from the consumption of VEGF inhibitors and from immunotherapy to prevent such catastrophic complications.

## Primary specialities involved other than cardiology

Vascular surgery, cardiovascular oncology, anaesthesia and intensive care.

## Introduction

Despite the promising effects seen with the combination of tyrosine kinase inhibitors (TKIs) with immune checkpoint blockade (ICB) in the management of metastatic renal cancer,^1^ acute aortic syndromes (AASs) are potential side effects of axitinib with pembrolizumab. This ICB induces inflammation in different body parts, including the blood vessels, which increases the risk of a person developing AAS.^2–4^ However, reports of an increased incidence of aneurysms and aortic dissection in patients receiving TKIs and ICB have been challenged.^2,3^ In this study, we report a well-documented case of a patient with acute intra-mural haematoma (IMH) following the consumption of axitinib and pembrolizumab that highlights and explores the link between TKIs, ICB, and AAS, including the mechanism, pharmacokinetics, and emergency management.

Our aim is to scrutinize the importance of cardiovascular oncology, determine the extent to which vascular endothelial growth factor receptor (VEGFR) inhibitors with immunotherapy impact cardiovascular function, and investigate the mechanisms underlying cardiovascular toxicities induced by such novel anti-angiogenic treatments.

## Summary figure

**Table:** 

271 days prior to the event	The patient presented with Stage 4 clear cell renal cell carcinoma and had a right radical nephrectomy.
120 days prior to the event	A suspicious-looking chest lesion on computed tomography angiography (CTA) with query metastasis.
63 days prior to the event	The patient received axitinib (5 mg b.i.d.) and pembrolizumab every 21 days.
1 day prior to the event	The patient received her third pembrolizumab cycle (240 mg i.v.).
Day 0 (event)	Acute illness with sharp intra-scapular pain 12 h after her third pembrolizumab cycle. Computed tomography angiography showed an intra-mural haematoma (IMH) >11 mm with a 33 mm dilated descending thoracic aorta.
Day 1	Vascular surgery referral. An interval 18 h CTA documented an increase in IMH to 14 mm with a 43 mm dilated descending aorta with an area of the free rupture and intractable pain not responding to opiates. A thoracic endovascular aortic repair was performed.
Day 2	The pain subsided and blood pressure normalized.
Day 3	A follow-up CTA showed total modulation of her aorta, and she was discharged home on clopidogrel plus angiotensin II inhibitors.
Day 90	A follow-up CTA demonstrated total aortic remodelling without stent graft–induced new entry (SINE).
Day 270	A follow-up CTA showed total disappearance of the perforation with a normal aortic contour without SINE.

## Case presentation

**Figure 1:**
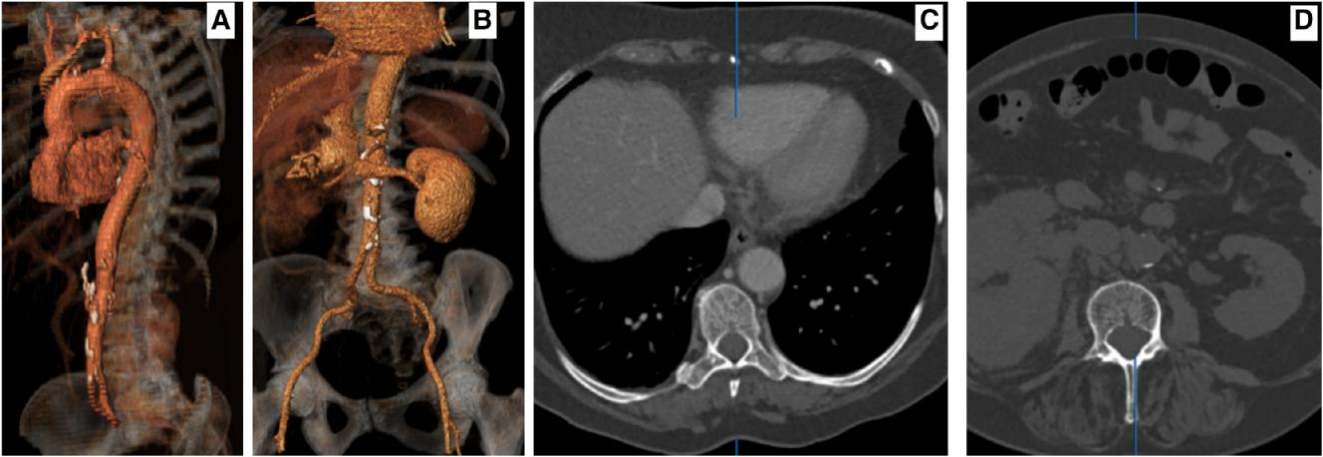
(*A*) Pre-operative to right nephrectomy, proximal three-dimensional imaging documenting a normal aorta. (*B*) Pre-operative to right nephrectomy, proximal three-dimensional imaging intravenous pyelogram revealing a tumour in the right kidney. (*C*) Pre-operative to right nephrectomy, axial computed tomography angiography documenting a normal aorta. (*D*) Pre-operative to right nephrectomy, axial intravenous pyelogram documenting a right kidney tumour.

A 73-year-old woman presented to the emergency department with sharp intra-scapular pain 12 h after her third pembrolizumab cycle (240 mg i.v.). She was on axitinib (5 mg b.i.d.) for 63 days and received pembrolizumab every 21 days for her Stage 4 clear cell renal cell carcinoma, diagnosed 9 months ago (*Figure 1A–D*).

On admission, her blood pressure was 200/95  mmHg, heart rate 54 b.p.m., respiratory rate 18 breaths/min, oxygen saturation 98% in room air, and temperature 36.1°C. Her cardiovascular, respiratory, and gastrointestinal system examinations were otherwise unremarkable.

Four months ago, she had a suspicious-looking chest lesion on computed tomography angiography (CTA) with query metastasis. Her haemoglobin was 10.5 g/dL (normal: 12–15 g/dL) and d-dimer 3000 ng/mL (normal: 0–500 ng/mL) with normal platelets, neutrophil count, and calcium.

She was on salazopyrin for 10 years for rheumatoid arthritis and angiotensin II inhibitors for hypertension. Given her history of rheumatoid arthritis, the oncology team recommended pembrolizumab and axitinib. All follow-up scans until her latest admission showed a normal aorta (*Figure 1A–C*).

**Figure 2:**
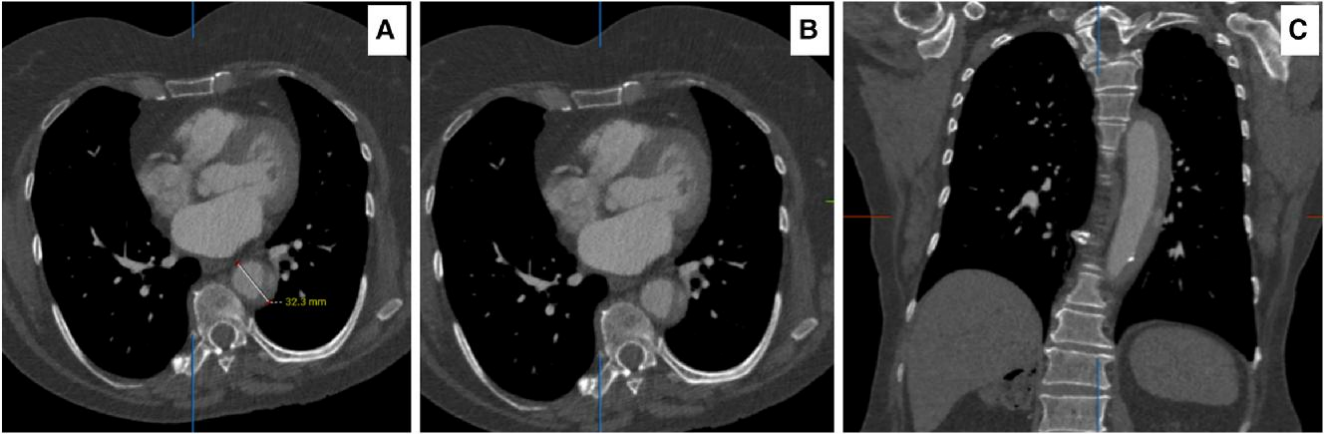
(*A*, *B*) Axial computed tomography angiography 18 h post tyrosine kinase inhibitors and immune checkpoint blockade, acute intra-mural haematoma of 32.3 mm at two levels. (*C*) Sagittal computed tomography angiography demonstrating the length of intra-mural haematoma with the active perforation.

**Figure 3:**
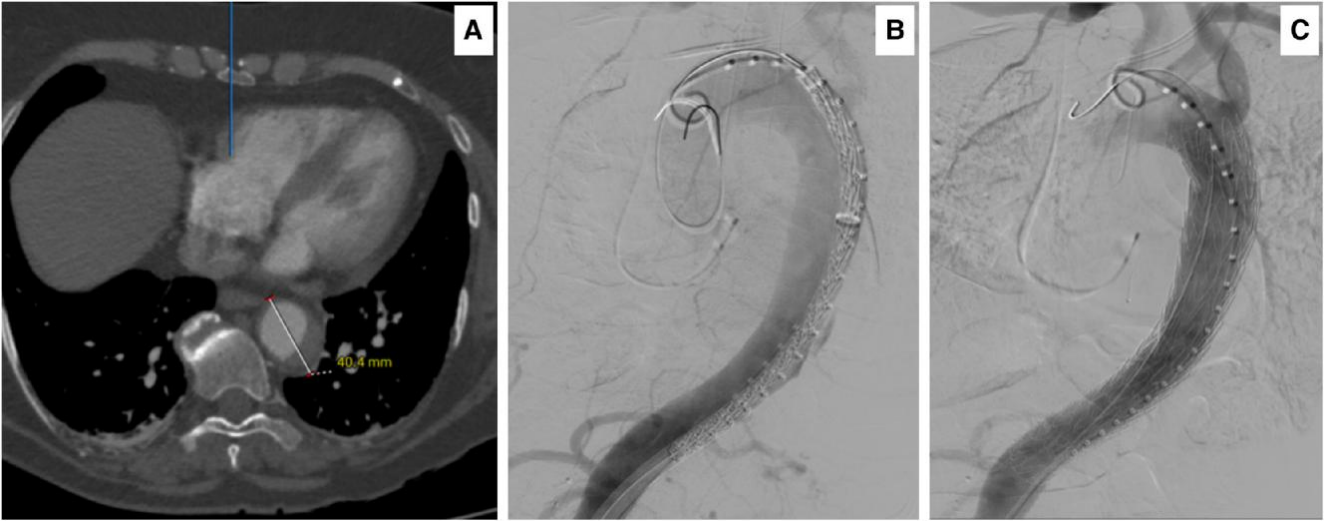
(*A*) Axial computed tomography angiography 18 h post admission demonstrated an increase in the size of acute intra-mural haematoma and aorta to 40.4 mm. (*B*) Intra-operative digital subtraction angiography, documenting the leak at the level of T9–T10 and CTAG in position before deployment. (*C*) Complete intra-operative digital subtraction angiography, documenting good positioning of the thoracic endovascular aortic repair CTAG graft and excluding the acute intra-mural haematoma. Note the patent left subclavian artery and celiac axis.

Computed tomography angiography on admission showed an IMH of >11 mm with a dilated descending thoracic aorta of 33 mm (*Figure 2A–C*). An interval 18 h CTA documented an increase in her IMH to 14 mm with a 43 mm dilated descending aorta with an area of free rupture (*Figure 3A–C*).

We employed the SPAU2 [S: **S**ystolic blood pressure (SBP), P: **P**ericardial effusion, A: **A**scending aortic diameter, U2: **U**lcer-like projection (ULP) in ascending aorta] risk score prediction for fatal events in acute type A IMH.^4^ The SPAU2 score assigns 1 point each for SBP <120 mmHg on arrival, pericardial effusion on admission CT, and ascending aortic diameter >45 mm on admission CT and 2 points for ULP in ascending aorta evident on admission CT. Our IMH recommendation is that if the score lies between 1 and 3 with continuous pain not controlled by opiates, we have a low threshold for intervention. In this study, our patient’s SPAU2 score was 1.^4^

She still had intractable pain, not responding to opiates, and her SBP was maintained below 120 mmHg with labetalol infusion.

On-table angiogram revealed a ruptured intima, following which an emergency thoracic endovascular aortic repair (TEVAR) was performed through the right groin using a conformable GORE® TAG® thoracic endoprosthesis (CTAG) device (W.L. Gore and Associates, Flagstaff, AZ, USA) 37–20, which sealed the rupture (*Figure 3C*).

**Figure 4:**
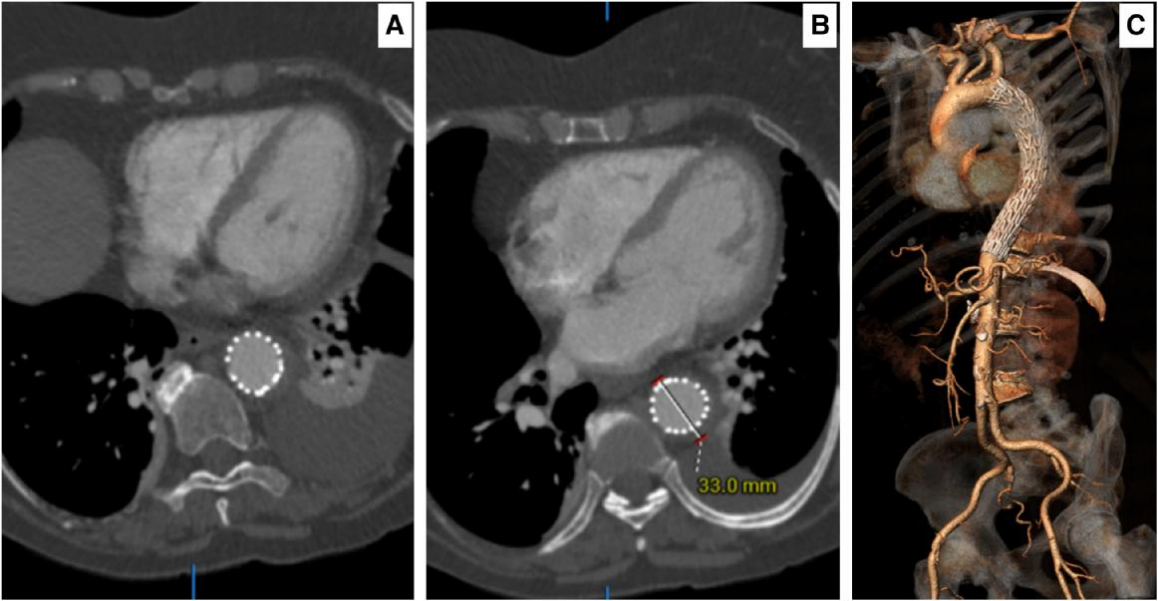
(*A*) A 96 h axial computed tomography angiography post intervention demonstrating total modulation of acute intra-mural haematoma. (*B*) A 96 h axial computed tomography angiography post intervention demonstrating a decrease in acute intra-mural haematoma size to 33 mm. (*C*) A 96 h three-dimensional computed tomography angiography reconstruction demonstrating no stent graft–induced new entry and patent great vessels of head and neck visceral branches with the stable aorta.

**Figure 5:**
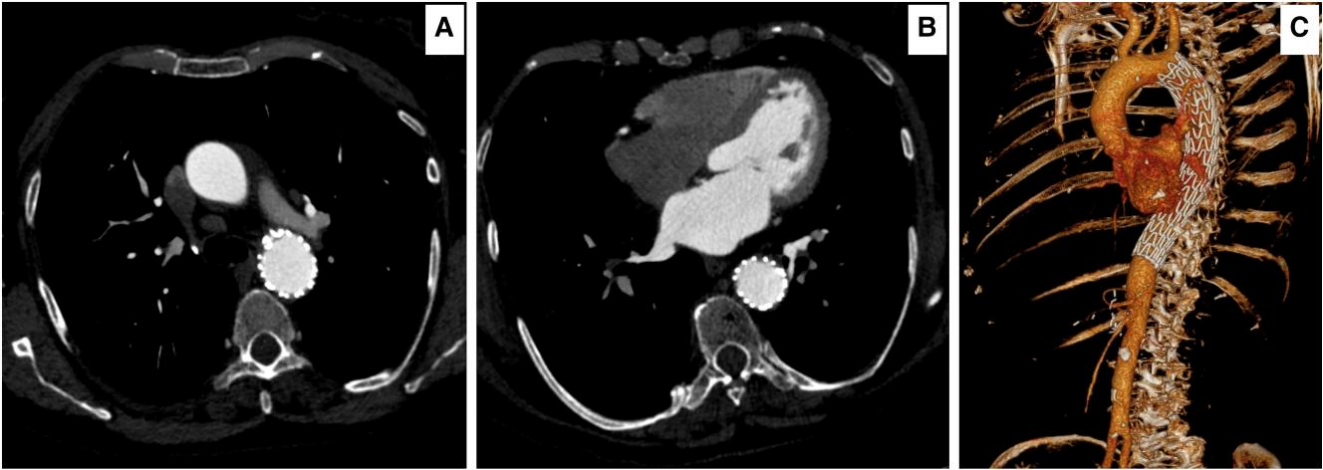
(*A*) A follow-up computed tomography angiography at 270 days post insult and thoracic endovascular aortic repair demonstrate total remodelling of the acute intra-mural haematoma with no evidence of endoleak and retrograde stent graft–induced new entry. (*B*) A follow-up computed tomography angiography at 270 days showing a completely ruptured acute intra-mural haematoma with a total resolution of the acute aortic catastrophe. (*C*) A three-dimensional reconstruction demonstrating patent celiac, both renal and the artery of Adamkiewicz (also arteria radicularis magna) at the level of the distal thoracic endovascular aortic repair, with no evidence of stent graft–induced new entry.

Post-operatively, the patient recovered fully without any neurological or cardiovascular issues. Her pain disappeared and her blood pressure normalized. She was advised to avoid the combination of TKI with ICB. Ninety-six hours of follow-up CTA showed total modulation of her aorta (*Figure 4A–C*). She was discharged home on clopidogrel plus angiotensin II inhibiters. Two hundred and seventy days of follow-up CTA demonstrated total aortic remodelling without stent graft–induced new entry (*Figure 5A–C*).

## Discussion

Axitinib is a targeted therapy for advanced kidney cancer and works by blocking the growth of new blood vessels and vasa vasorum that feed tumours. Axitinib inhibits the activity of tyrosine kinases, which are enzymes that play a crucial role in cellular signalling pathways involved in cell growth, differentiation, and survival. However, tyrosine kinases are involved in maintaining the integrity of the vascular system.^5–7^ They regulate the contractility of smooth muscle cells and the production of extracellular matrix (ECM) proteins, which are critical arterial wall components.

Axitinib increases the risk of a person developing high blood pressure, cardiovascular complications, and AAS.^2–4^ Tyrosine kinase inhibitors can lead to weakened arterial walls and an increased risk of aneurysm formation and aortic dissection. Tyrosine kinase inhibitors have been shown to reduce the expression of genes encoding for collagens, which are the main structural proteins of the arterial wall.^6,7^ This reduction in collagen production will weaken the arterial wall.

Pembrolizumab is an immune checkpoint blocker that blocks the interaction between programmed cell death protein 1 (PD-1) and its ligands, PD-L1 and PD-L2, leading to enhanced T-cell activation and immune response against cancer cells. An important caveat is that T-cell activation extends beyond cancer cells to normal tissues and organs, including the arterial wall. Collagen and elastin are critical components of the arterial wall’s ECM, providing strength and elasticity to the blood vessels.^7^ The destruction of collagen and elastin in the arterial wall will weaken and rupture the vessel wall, leading to AAS, aneurysms, and dissections.

The introduction of novel anti-cancer treatments targeting VEGFRs has changed the therapeutic approach in oncology. However, unanticipated cardiovascular complications emerged with their introduction into clinical practice, and these included hypertension, left ventricular dysfunction, and thromboembolism.^3,8,9^ Even though these therapeutics strongly affect haemodynamic balance in patients, the pathophysiological mechanisms by which they impair the cardiovascular function are still largely unknown, leading to a reduction of therapeutic dosage or a temporary or permanent treatment interruption.

The most catastrophic complication of AAS, particularly IMH and acute type B aortic dissection, has a 21% rate of incidence of acute aortic rupture.^4^ We employed the ‘Time Until Treatment Equipoise’ strategy, which is the point in time during follow-up, after which an intervention is most beneficial, as the mortality risk of intervention is lower than the mortality risk of continuing current medical management.

Analyses of pulse wave velocity in patients with cancer treated with TKIs have revealed the haemodynamic effects of these drugs on arterial stiffness, and axitinib could detrimentally and independently increase aortic stiffness and cause arteriosclerosis.^9,10^

In the aortic wall, the VEGF is predominantly expressed in the smooth muscle cells in vessel walls and is highly expressed in atherogenic lesions.^11^ The VEGF localized in inflammatory cells in atherosclerotic lesions plays inflammatory roles rather than angiogenic roles in the large vessel walls.^11,12^ As VEGF signalling is vital to the maintenance of vessel walls, axitinib possibly contributes to the vulnerability of the aortic wall by inhibiting it. The balance between collagen synthesis and degradation is important for the elasticity and vulnerability of vessel walls. Recent studies have demonstrated that matrix metallopeptidase 9 (MMP-9) over-expression is associated with various vascular diseases, including aortic dissection.^13^ Matrix metallopeptidase 9 is produced from fibroblasts, smooth muscle cells, inflammatory neutrophils, and macrophages in the vessel wall and is thought to play an important role in the onset of aortic dissection.^14^

We reviewed reports of VEGF inhibitor–associated aortic dissection and found that not all cases describing TKI-induced aortic dissection reported that patients had hypertension before the onset of aortic dissection, which was consistent with the patient case that we reported.^2,3,9,15^ Based on this fact, we speculated that the VEGF–VEGFR signal pathway with ICB plays an important role in AAS in addition to hypertension.^15^

Our experience mirrors others of failed medical treatment in IMH with higher mortality than expected, as patients with aortic dissection/IMH/penetrating aortic ulcer have a significant risk of aortic death in the first 14 days from diagnosis.^4^ Patients who survive the initial event without surgery have a 10-fold higher risk of subsequent aortic intervention. In the case of our patient, we found that she had a non-resolving IMH, and she had signs of high-risk IMH that could progress to pan-aortic dissection and rupture given her haematoma thickness >10–13 mm on maximum aortic diameter. Furthermore, a high SPAU2 score (>3) requires immediate intervention.^4,16^ However, IMH with early precipitous aortic growth strongly indicates an unfavourable outcome. It must be considered a warning to execute elective intervention, and in our patient, we intervened as her SPAU2 score was 1.

The exact risk of a person developing aortic dissection and aneurysm after treatment with axitinib and immune checkpoint inhibition is not well established and may vary depending on individual patient factors.^17,18^ However, it is essential for patients receiving such treatments to be closely monitored for any signs of cardiovascular complications, including chest pain, shortness of breath, or sudden onset of back pain. If any of these symptoms occur, patients should seek medical attention immediately. Since patients included in the pre-hypertension category (SBP from 120 to 139 mmHg and/or diastolic blood pressure from 80 to 89 mmHg) suffer a higher incidence of both cardiac and vascular events, and acute aortic dissection (AAD) is a life-threatening medical emergency associated with high rates of morbidity and mortality, clinicians must be aware of such events and complications, and hence, tighter grading and closer surveillance of blood pressure are highly recommended.

Our cases have identified a potential safety concern associated with the combination of ICB with axitinib, which is the development of aortitis, aneurysms, IMH, and AAS. Our findings raise concern in selected patients for the potential implications of discontinuing tyrosine inhibitors added to ICB. Future research may elucidate the underlying mechanisms of this safety concern and identify strategies for mitigating its risks. Our study suggests that this may occur through a mechanism involving the induction of hypertension by oral axitinib, as well as MMP-9 degradation and the intravenous pembrolizumab destruction of elastin and collagen in the aortic wall.

A pharmacovigilance investigation by the US Food and Drug Administration Adverse Event Reporting System database reported an odds ratio for the association between VEGF inhibitor and AAS.^19^ Among them, ramucirumab was associated with a higher reporting odds ratio of aneurysm and artery dissection (3.68, 95% confidence interval 2.18‒6.23) with a statistically significant difference in onset (*P* < 0.001). The median time to aneurysm and artery dissection was 79.5 days, with more cases in patients aged 45–74. Finally, VEGF inhibitor led to 19.98% of deaths and 29.81% of hospitalizations.

Hypertension, proteinuria, and AAS are the most severe adverse effects of anti-VEGF drugs.^3,9^ However, no pharmacovigilance study exists to explore the relationship between VEGF inhibitor–mediated AAD with ICB and the knowledge of vascular safety profile. All remain poorly represented in clinical practice.^19^

However, there is a spectrum of immune-related adverse events (irAEs) that are different from those of conventional chemotherapy that is necessary to keep in mind during ICB treatment. Although aortitis is uncommon, these irAE-related fatal toxicities require prompt recognition. The occurrence of these complications requires a stoppage of the treatment. The choice to retreat depends on multiple factors, including the severity and nature of the initial irAE and the success of toxicity resolution with immunosuppression.^20^

The axitinib-induced hypertensive response is associated with regionally selective vasoconstrictions. Clinicians should be aware of this life-threatening complication related to using TKIs with ICB. Takotsubo cardiomyopathy–like myocardial dysfunction is associated with both modalities of treatment.

## Conclusion

Tyrosine kinase inhibitors with ICB increase the risk of AAS by inhibiting pathways involved in maintaining the integrity of the arterial wall. The incidence of these events varies depending on patient factors, and clinicians need to be aware of this potential risk when prescribing both drugs. Regular monitoring and early intervention are necessary to manage these potentially life-threatening events.

## Data Availability

The data underlying this article are available in the article and in its online supplementary material.

## Lead author biography

Prof. Sherif Sultan is a pioneering vascular/endovascular surgeon and the founder of the Western Vascular Institute, Ireland, a charitable research foundation committed to vascular research, technical innovation, and education. He is the current president of the International Society of Vascular Surgery and the American Society of Angiology, Irish chapter. He had pioneered the techniques of subintimal angioplasty, DRESS technique for complex EVAR, kinetic elephant trunk for pan-aortic dissection, TIGER protocol for infra-diaphragmatic aortic dissection, triple neuroprotection for patients with acute vascular stroke post the 24 h opportunity window, and art-assist for critical limb ischaemia patients.

**Consent:** The authors confirm that written consent for submission and publication of this case report, including images and associated text, has been obtained from the patient in line with COPE guidance. All patients have consented to publish this case report, including the images.

**Funding:** This research received no specific grant from any funding agency in the public, commercial, or not-for-profit sector.
